# Cultural adaptation and psychometric evaluation of the Kinyarwanda version of the problem areas in diabetes (PAID) questionnaire

**DOI:** 10.1186/s12955-021-01821-w

**Published:** 2021-07-22

**Authors:** Charilaos Lygidakis, Jean Paul Uwizihiwe, Michela Bia, Per Kallestrup, Damas Dukundane, Brenda Asiimwe-Kateera, Simon Pierre Niyonsenga, Claus Vögele

**Affiliations:** 1 Department of Behavioural and Cognitive Sciences, University of Luxembourg, Porte des Sciences 11, 4366 Esch-sur-Alzette, Luxembourg; 2 College of Medicine and Health Sciences, University of Rwanda, Huye, Rwanda; 3 Centre for Global Health, Department of Public Health, Aarhus University, Aarhus, Denmark; 4 Luxembourg Institute of Socio-Economic Research (LISER), Esch-sur-Alzette, Luxembourg; 5 Butaro Cancer Centre of Excellence, Burera, Rwanda; 6 AIDS Healthcare Foundation (AHF) Rwanda, Kigali, Rwanda; 7 Rwanda Biomedical Center, Kigali, Rwanda

**Keywords:** Problem areas in diabetes, PAID, Psychometrics, Validation study, Diabetes mellitus, Rwanda, Africa South of the Sahara

## Abstract

**Background:**

High prevalence rates in diabetes-related distress have been observed in several studies; however, in the region of Sub-Saharan Africa evidence is lacking as is, for example, the case for Rwanda, where diabetes prevalence is expected to increase over the next decade. The aim of this study is to report on the translation and cultural adaption of the problem areas in diabetes (PAID) questionnaire into Kinyarwanda and its psychometric properties.

**Methods:**

The questionnaire was translated following a standard procedure. Interviews were conducted with 29 participants before producing a final version. For the psychometric evaluation, a sample of 266 patients with diabetes mellitus, aged 21–64 years old were examined. Participants either came from a separate cluster-randomised controlled trial or were recruited ad-hoc for this study. The evaluation included testing internal consistency, known groups validity, and construct validity. A series of confirmatory factor analysis were conducted investigating seven previously established factorial structures. An exploratory factor analysis (EFA) was also carried out to examine the structure further.

**Results:**

The full scale showed good internal reliability (Cronbach’s α = 0.88). A four-factor solution previously tested in Spain with subdimensions of emotional, treatment, food-related and social-support problems demonstrated adequate approximate fit (RMSEA = 0.056; CFI = 0.951; TLI = 0.943). The EFA revealed a four-factor structure; however, two of these factors were not as homogeneous and easily interpretable as those of the Spanish model.

**Conclusions:**

The psychometric properties of the Kinyarwanda version of PAID are acceptable. The questionnaire can be helpful in research and clinical practice in Rwanda, however certain cross-cultural differences should be taken into account.

## Background

Psychological distress refers to a range of emotional response to stressors [1, 2]. It is experienced as the inability of effective coping, and is manifested by a change in emotional status, discomfort, communication of discomfort, and harm [1]. Patients with diabetes often report psychological distress as they need to cope with the burden of chronic treatment, self-care and self-management, and may experience lack of social support, fear of complications or hypoglycaemia, and powerlessness [3–5]. High prevalence rates in diabetes-related distress have been observed in several studies [6] and have been associated with poor glycaemic control, self-care, and quality of life [7, 8]. A recent meta-analysis of studies in patients with type 2 diabetes mellitus estimated its prevalence at 36% [4], while Fisher et al. [9] reported rates of psychological distress in 42.1% of patients with type 1 diabetes mellitus. Nevertheless, studies in Sub-Saharan Africa are lacking [10–12], as is, for example, the case for Rwanda, where age-adjusted comparative diabetes prevalence in adults 20–79 years has been estimated at 5.1% [13].

Increasing awareness of diabetes distress can help in researching specific interventions and its management in clinical settings. The problem areas in diabetes (PAID) questionnaire is a broadly employed self-report measure, which has been shown to be significantly associated with glycaemic control, adherence to treatment and complications [14], and to be linked to other associated constructs (e.g., general emotional distress, depression) [14, 15]. It has been translated in many languages, has demonstrated high internal reliability and good responsiveness to change, and has been employed in a wide range of interventions [14–18].

The aim of this paper was to report on the translation and cultural adaption of the PAID questionnaire into Kinyarwanda and evaluate its psychometric properties.

## Material and methods

### The problem areas in diabetes (PAID) questionnaire

The questionnaire consists of 20 items representing different diabetes-related issues that may be a problem for the patient. Each item can be answered using a five-point Likert scale ranging from 0 (not a problem) to 4 (serious problem). The individual items can be summed up and multiplied by 1.25 to compute a total score, which ranges between 0 and 100, with higher scores indicating more severe distress [15].

A one-factor structure was originally proposed [14], however cross-cultural adaptations have revealed its multidimensional nature [8, 18–24]. The constructs of some of the models are presented in Table 1.

**Table 1:**
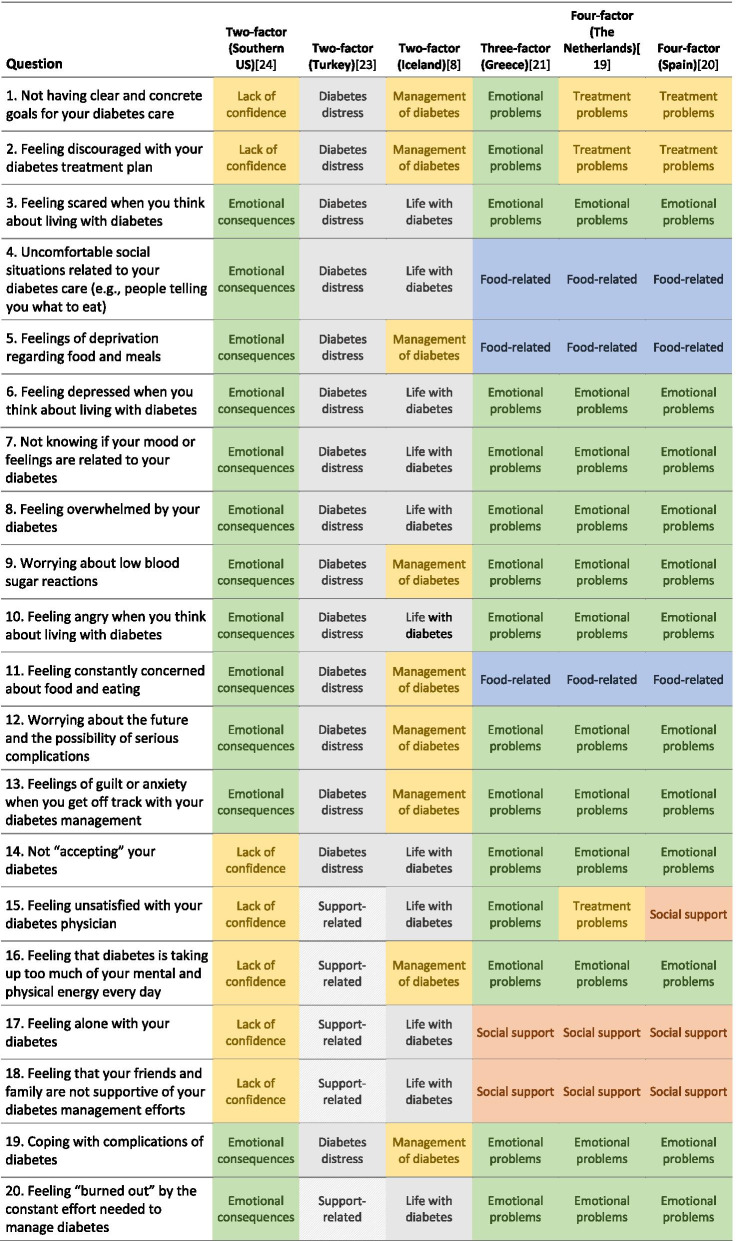
Constructs of the previously established models of PAID

The different colours represent similarly identified constructs and highlight how they differ across the various translations

### Translation and cross-cultural adaptation

For the translation of the questionnaire, a standard protocol was followed [25]:

- Two native Rwandans, proficient in English, translated the questionnaire into Kinyarwanda independently, following an item intent guide.
- The two translations were synthesised into one, addressing any discrepancies.
- Two English native speakers with excellent language skills in Kinyarwanda back-translated the questions into English, while blinded to the original version.
- Subsequently, the two backward translations were reconciled into one.
- All versions of the questionnaires were evaluated by an expert panel, consisting of the two Rwandan forward translators, one of the backward translators, an epidemiologist, a local bilingual representative, and the two researchers conducting the study. The aim was to appraise the results of the translations, evaluate their semantic, idiomatic, experiential and conceptual equivalence, and produce a prefinal version.
- A report was prepared providing an account of these steps, the controversial items and the ways they were resolved in the consensus translation. The report and the prefinal version were shared with the questionnaire developer, and consent was received.

The prefinal version was assessed with interviews using a sample of patients with diabetes mellitus. The objective was to evaluate: the comprehension of the translated questions and the answer categories; whether respondents could retrieve relevant information from memory; the effort requested to answer; the degree of interest; and social desirability bias. The interviews were conducted in rounds. To attain maximum variability of the participants, the interviews were conducted in four different hospitals. After each round, modifications were proposed for some items based on the interview transcripts and notes. A new iteration of the questionnaire was then prepared and tested in the following round. Lastly, a final version was produced and a report was made available to the developer.

### Psychometric evaluation and statistical analysis

#### Participants

The psychometric evaluation was initially designed as part of a cluster-randomised controlled trial (RCT) that aimed at determining the efficacy of an integrated mobile-health and community-health-worker programme for the management of diabetes in primary health care in Rwanda (ClinicalTrials.gov registration: NCT03376607) [26]. Patients living with diabetes were recruited in outpatient clinics for non-communicable diseases in seven out of nine hospitals of the trial between January and August 2019: Bushenge, Kibungo, Kibuye, Kinihira, Muhima, Ruhengeri, and Rwamagana. For the psychometric evaluation of the questionnaire, we used data exclusively from the baseline assessment of the patients participating in the RCT.

For the purpose of conducting the confirmatory factor analysis (CFA), at least 200 participants would be necessary [20, 22, 27, 28]. The power analysis of the RCT indicated a sample size of 324 participants, which was also adequate for conducting the CFA. Nonetheless, the pre-enrolment screening revealed that a sufficient number of patients living with diabetes could not be recruited in the specific recruitment areas selected for the RCT [26]. Furthermore, logistical challenges impeded the prompt activation of the last two of the nine hospitals (Kabutare and Ruhango).

For these reasons, we recruited an additional sample specifically for the purposes of the psychometric evaluation. This supplementary cohort consisted of patients residing in additional zones in the catchment areas of same hospitals, except for the hospitals of Kibungo, Kibuye and Kinihira, where the number of patients was particularly low. The recruitment was carried out between June and December 2019. Both samples followed the same inclusion criteria: patients aged 21–80 years, and diagnosed with diabetes mellitus at least six months prior to study onset. Exclusion criteria for both samples were: illiteracy, severe hearing or visual impairments, severe mental health conditions, pregnancy or post-partum period. The classification of diabetes type was based on the patients’ clinical records available at the hospitals.

As older people may present more multimorbidity which can impact emotional distress levels, only data from participants between 21 and 64 years old were included in the final sample for analysis [18]. As the precise date of diagnosis of diabetes was unknown for some participants, only those with at least one year of diagnosis were included so as to limit the effect of the emotional distress linked to recent diagnosis [18, 29].

#### Statistical analyses

Statistical analyses were performed on Stata version 16 and Mplus version 7. To assess construct validity, a series of CFA were conducted using previously established structures:

1. Two-factor model (rural African American women with type 2 diabetes mellitus in a Southern state of the US) [24]
2. Two-factor model (insulin-naïve type-2-diabetes-mellitus patients in Turkey) [23]
3. Two-factor model (insulin-dependent patients in Iceland) [8]
4. Three-factor model (patients with type 2 diabetes mellitus in Greece) [21]
5. Four-factor model (The Netherlands) [19]
6. Four-factor model (Spain) [20]

For comparison, the one-factor model of the original study was also fitted to the data [14, 15]. The factor structure of the Swedish version was not tested, as the authors replaced one item (*“﻿coping with complications of diabetes”*) with a new one (*“feeling unsatisfied with your diabetes specialist nurse”*) to match the local health system [22].

The weighted least square mean and variance adjusted (WLSMV) estimator was used in the CFA, which is considered more appropriate for ordinal data [30]. The root mean square error of approximation (RMSEA), the comparative fit index (CFI), and the Tucker Lewis index (TLI) were used to examine the approximate model fit. For RMSEA, values of less than 0.05 were indicative of a close fit and those between 0.05 and 0.08 were interpreted as adequate fit [31, 32]. The 90% confidence intervals of RMSEA were also evaluated, as they should be less than 0.05 for the lower bound and no worse than 0.08 for the upper one [31]. For CFI and TLI, values of 0.90 and above were regarded as acceptable fit [31, 32]. Hu’s and Bentler’s recommendation of raising such cut-offs to 0.95 was also taken into account [31, 33]. The relative χ^2^ was also calculated and a value of 2 or less was deemed adequate [31]. Finally, although the weighted root mean-square residual (WRMR) was computed and values of 1 or lower were considered a good fit, the experimental nature of this statistic thwarted drawing conclusions based on it [31, 32, 34]. A further evaluation of the structure was performed with an exploratory factor analysis (EFA) using the WLSMV estimator and Geomin rotation [35].

To assess internal reliability Cronbach’s α and composite reliability were calculated. Mean differences in total score and in the scales of the model with the closest fit were investigated across socio-demographic and clinical groups with the Mann–Whitney U test (between two groups) and the Kruskal–Wallis test (between three or more groups), applying the Bonferroni correction in post-hoc tests for planned contrasts. This non-parametric approach was adopted as the total scale scores were derived from ordinal variables. Effect sizes were calculated based on *z* values; *r* of 0.10, 0.30 and 0.50 were interpreted as small, medium and large effects respectively [36]. For continuous variables, Spearman’s correlation was used to determine which of them were associated with the total score and scales. Correlation coefficients below 0.4 were considered as weak, those between 0.4 and 0.7 as moderate, and those above 0.7 as strong [37, 38].

### Ethical approval

Ethical approval was obtained from the Rwanda National Ethics Committee (100/RNEC/2017; renewed in 113/RNEC/2018 and 192/RNEC/2019; amended in 463/RNEC/2017 and 688/RNEC/2019), and the Ethics Review Panel of the University of Luxembourg (ERP 17-014 D2Rwanda; amended in ERP 17-048 D2Rwanda).

## Results

### Cultural adaptation

The expert panel evaluated all translations and reached a consensus, particularly with regard to the items without precise translation into Kinyarwanda: “feeling depressed” (item 6), “mood” (item 7), “taking up energy” (item 16), “coping with” (item 19), “burned out” (item 20). Three rounds of interviews were conducted thereafter with a total of 29 participants with diabetes: 18 women and 11 men, with median age of 48.5 (range 31–67), and median education of 6 years (range 0–12). Comprehension of the translated items was good and minor amendments were made to increase clarity and resolve any ambiguities. As there was no alternative way to express the concept of “mood”, item 7 was modified (final back translation: *“not knowing whether the way you feel in yourself is caused by your diabetes”*).

### Characteristics of the sample of the psychometric evaluation

Two hundred and five participants were included from the RCT and 122 were recruited additionally for the purposes of the evaluation. Of these, our analyses focused on data from the 266 participants who were 21–64 years of age and with at least one year after diagnosis of diabetes. All participants were of Rwandan nationality and spoke Kinyarwanda. The sample characteristics are presented in Table 2. The two samples (RCT and additional cohort) did not differ significantly in terms of age, years of completed education, area of residency, living situation, employment type, type of diabetes and years since the diagnosis of diabetes. The mean total score of PAID for the sample was 48.21 (SD = 18.83) and the median was 47.5 (IQR = 36.25–61.25).

**Table 2:** Sample characteristics

Gender, n (%)	
Female	181 (68.05)
Male	85 (31.95)
Age, mean (SD), median (IQR)	48 (10.92), 49 (40–57)
Marital status, n (%)	
Single	25 (9.40)
Married	152 (57.14)
Cohabitation	44 (16.54)
Divorced	4 (1.50)
Widowed	36 (13.53)
Other	5 (1.88)
Most usual living situation, n (%)	
Lives alone	6 (2.27)
Has other people living with him/her	258 (97.73)
Number of people are living with him/her, mean (SD), median (IQR)	4.97 (2.28), 5 (3–6)
Area of residency, n (%)	
Urban	80 (30.30)
Semiurban	65 (24.62)
Rural	119 (45.08)
Years of completed education, mean (SD), median (IQR)	7.59 (3.45), 6 (6–9)
Highest degree obtained, n (%)	
No formal education	18 (6.87)
Primary school	160 (61.07)
Secondary school	48 (18.32)
University degree	12 (4.58)
Vocational school	23 (8.78)
Postgraduate studies	1 (0.38)
Employment status, n (%)	
Unemployed	121 (45.49)
Employed	138 (51.88)
Retired	7 (2.63)
Abilities, mean (SD), median (IQR)^*a*^	
Writing	3.29 (0.66), 3 (3–4)
Read and understand	3.27 (0.67), 3 (3–4)
Converse with other people and understand	3.54 (0.54), 4 (3–4)
Hear clearly	3.59 (0.54), 4 (3–4)
See things clearly	3.12 (0.72), 3 (3–4)
Do normal daily activities	3.15 (0.76), 3 (3–4)
Move about the community by himself/herself	3.61 (0.56), 4 (3–4)
Self-rated overall health, mean (SD), median (IQR)^b^	3.27 (0.60), 3 (3–4)
Types of diabetes, n (%)	
Type I	26 (9.92)
Type II	228 (87.02)
Unknown	8 (3.05)
Years since diagnosis, mean (SD), median (IQR)	5.83 (4.67), 5 (2–8)

*SD* standard deviation, *IQR* interquartile range

^a^The abilities were evaluated using a four-point Likert scale ranging from 1 (cannot do at all) to 4 (can do very well)

^b^The overall health was evaluated using a five-point Likert scale ranging from 1 (very poor) to 5 (very good)

### Construct validity (confirmatory factor analysis)

The χ^2^ of all models was significant and, therefore, we examined the approximate fit indices. The four-factor model from Spain showed the best fit with adequate-to-good RMSEA, CFI and TLI (Table 3).

**Table 3:** Comparison of the different factor structures in the sample of under 65 years of age

Model	χ^2^	df	Relative χ^2^	RMSEA (90% CI)	CFI	TLI	WRMR
One-factor (Original US)	370.838	170	2.18	0.067 (0.058–0.076)	0.928	0.919	1.085
Two-factor (Southern US)	354.683	169	2.10	0.065 (0.055–0.074)	0.933	0.925	1.054
Two-factor (Turkey)	349.735	169	2.07	0.064 (0.054–0.073)	0.935	0.927	1.041
Two-factor (Iceland)	364.193	169	2.15	0.066 (0.057–0.076)	0.930	0.921	1.074
Three-factor (Greece)	327.065	167	1.96	0.060 (0.051–0.070)	0.942	0.934	1.003
Four-factor (The Netherlands)	307.809	164	1.88	0.058 (0.048–0.068)	0.948	0.940	0.962
Four-factor (Spain)	300.228	164	1.83	0.056 (0.046–0.066)	0.951	0.943	0.946

*df* degrees of freedom, *RMSEA* root mean square error of approximation, *CFI* comparative fit index, *TLI* Tucker Lewis index, *WRMR* weighted root mean-square residual

In the four-factor Spanish model, standardised factor loadings ranged from 0.451 to 0.762 for the emotional-problems scale; from 0.691 to 0.722 for the treatment-problems scale; from 0.485 to 0.689 for the food-related-problems scale; and from 0.536 to 0.838 for the social-support-problems scale (Table 4). Especially for the scales concerning food-related and﻿ emotional problems, the majority of the items did not load highly on their factors. Inter-factor correlations ranged from 0.500 to 0.730 showing sufficient discriminant validity, with the exception of the food-related and emotional-problems scales (inter-factor correlation = 0.906).

**Table 4:** Psychometric properties of the Kinyarwanda version of PAID, replicating the four-factor Spanish model

	Mean (SD), median (IQR)	Standardised loading ^a^	S.E	R^2^
*Emotional problems* (CR = 0.875, Cronbach’s α = 0.85)				
3. Feeling scared when you think about living with diabetes	2.27 (1.40), 2 (1–4)	0.709	0.037	0.503
6. Feeling depressed when you think about living with diabetes	2.08 (1.45), 2 (1–3)	0.762	0.028	0.581
7. Not knowing if your mood or feelings are related to your diabetes	2.12 (1.23), 2 (1–3)	0.481	0.047	0.231
8. Feeling overwhelmed by your diabetes	2.39 (1.33), 2 (1–4)	0.659	0.041	0.435
9. Worrying about low blood sugar reactions	2.33 (1.34), 2 (1–4)	0.451	0.047	0.204
10. Feeling angry when you think about living with diabetes	1.44 (1.43), 1 (0–3)	0.661	0.039	0.437
12. Worrying about the future and the possibility of serious complications	3.11 (1.15), 4 (3–4)	0.633	0.046	0.401
13. Feelings of guilt or anxiety when you get off track with your diabetes management	2.31 (1.20), 3 (1–3)	0.460	0.050	0.212
14. Not “accepting” your diabetes	1.27 (1.33), 1 (0–2)	0.556	0.047	0.309
16. Feeling that diabetes is taking up too much of your mental and physical energy every day	2.16 (1.38), 2 (1–3)	0.637	0.044	0.405
19. Coping with complications of diabetes	2.26 (1.30), 2 (1–3)	0.497	0.051	0.247
20. Feeling “burned out” by the constant effort needed to manage diabetes	1.88 (1.43), 2 (1–3)	0.737	0.032	0.544
*Treatment problems* (CR = 0.666, Cronbach’s α = 0.63)				
1. Not having clear and concrete goals for your diabetes care	1.99 (1.37), 2 (1–3)	0.722	0.064	0.521
2. Feeling discouraged with your diabetes treatment plan	1.71 (1.43), 2 (0–3)	0.691	0.061	0.478
*Food-related problems* (CR = 0.602, Cronbach’s α = 0.54)				
4. Uncomfortable social situations related to your diabetes care (e.g., people telling you what to eat)	1.71 (1.31), 2 (1–3)	0.556	0.049	0.309
5. Feelings of deprivation regarding food and meals	2.01 (1.35), 2 (1–3)	0.485	0.052	0.235
11. Feeling constantly concerned about food and eating	2.38 (1.39), 3 (1–4)	0.689	0.047	0.475
*Social support problems* (CR = 0.745, Cronbach’s α = 0.61)				
15. Feeling unsatisfied with your diabetes physician	0.54 (1.14), 0 (0–0)	0.536	0.077	0.287
17. Feeling alone with your diabetes	1.25 (1.36), 1 (0–2)	0.838	0.044	0.702
18. Feeling that your friends and family are not supportive of your diabetes management efforts	1.38 (1.46), 1 (0–3)	0.718	0.045	0.516

*SD* standard deviation, *IQR* interquartile range, *CR* composite reliability

^a^All standardised loadings were found significant (*p* < 0.001)

Associations between socio-demographic and clinical variables and the questionnaire are presented in Table 5. Age and gender had no impact on the PAID score, while educational level and area of residency showed a small effect. There was an inverse, albeit weak, correlation between self-rated overall health and all distress scales, but not for the treatment-problems scale. There were no differences between the types of diabetes, and the duration of the disease was only weakly correlated with the social-support-problems scale. Scores on this scale, however, did not differ across the groups of patients with different years of diabetes duration.

**Table 5:** Relationships between socio-demographic and clinical variables and PAID

	Emotional problems^a^ (range 0–60)	Treatment problems^a^ (range 0–10)	Food-related problems^a^ (range 0–15)	Social support problems^a^ (range 0–15)	Total score^a^ (range 0–100)
*Gender*					
Female, *median (IQR)*	32.5 (23.75–41.25)	5 (2.5–7.5)	7.5 (5–11.25)	2.5 (1.25–7.5)	47.5 (36.25–62.5)
Male, *median (IQR)*	31.25 (23.75–41.25)	3.75 (2.5–6.25)	7.5 (5–8.75)	2.5 (1.25–6.25)	47.5 (33.75–60)
Mann–Whitney test	z = 0.227, *p* = 0.821	z = 1.167, *p* = 0.244	z = 0.630, *p* = 0.530	z = 0.589, *p* = 0.557	z = 0.737, *p* = 0.462
ES	r = 0.014	r = 0.072	r = 0.039	r = 0.036	r = 0.045
*Age*, *Spearman’s correlation*	r_s_ = − 0.069 *p* = 0.263	r_s_ = 0.017 *p* = 0.780	r_s_ = 0.072 *p* = 0.244	r_s_ = − 0.075 *p* = 0.224	r_s_ = − 0.046 *p* = 0.451
*Age*					
21–44 years, *median (IQR)*	32.5 (25–42.5)	3.75 (2.5–6.25)	6.25 (5–10)	3.75 (1.25–7.5)	48.75 (36.25–61.25)
45–54 years, m*edian (IQR)*	31.25 (21.25–41.25)	5 (1.25–6.25)	8.75 (5–11.25)	2.5 (0–5)	47.5 (35–63.75)
55–64 years, *median (IQR)*	31.25 (23.75–41.25)	5 (2.5–7.5)	7.5 (5–11.25)	2.5 (1.25–6.25)	46.25 (33.75–60.625)
Kruskal Wallis H	H(2) = 0.935, *p* = 0.627	H(2) = 0.493, *p* = 0.782	H(2) = 1.414, *p* = 0.493	H(2) = 4.118, *p* = 0.128	H(2) = 0.815, *p* = 0.665
*Area of residency*					
Urban, *median (IQR)*	29.375 (18.75–39.375)	3.75 (1.25–6.25)	6.25 (5–10)	2.5 (0–6.875)	45 (26.875–56.875)
Rural or semi, *median (IQR)*	32.5 (25–42.5)	5 (2.5–7.5)	7.5 (5–10)	2.5 (1.25–6.25)	49.375 (37.5–63.125)
Mann–Whitney test	z = − 2.211, *p* = 0.027	z = − 1.684 *p* = 0.092	z = − 1.355, *p* = 0.176	z = − 1.612, *p* = 0.107	z = − 2.133, *p* = 0.033
ES	r = − 0.136	r = − 0.104	r = − 0.083	r = − 0.099	r = − 0.131
*Years of completed education, Spearman’s correlation*	r_s_ = − 0.214 *p* = 0.001	r_s_ = − 0.203 *p* = 0.001	r_s_ = − 0.115 *p* = 0.061	r_s_ = − 0.214 *p* = 0.001	r_s_ = − 0.240 *p* < 0.001
*Highest degree obtained*					
No formal education or primary school, *median (IQR)*	33.75 (26.25–43.75)	5 (2.5–7.5)	7.5 (5–11.25)	3.75 (1.25–7.5)	51.25 (41.25–63.75)
Secondary school, university, vocational school or postgraduate, *median (IQR)*	26.875 (20–35.635)	3.75 (1.25–6.25)	7.5 (5–8.75)	2.5 (0–5)	41.25 (30–52.5)
Mann–Whitney test	z = 3.777, *p* < 0.001	z = 3.406, *p* = 0.001	z = 1.635, *p* = 0.102	z = 2.183, *p* = 0.029	z = 3.818, *p* < 0.001
ES	r = 0.233	r = 0.210	r = 0.101	r = 0.135	r = 0.236
*Self-rated overall health, Spearman’s correlation*	r_s_ = − 0.245 *p* < 0.001	r_s_ = − 0.078 *p* = 0.205	r_s_ = − 0.183 *p* = 0.003	r_s_ = − 0.306 *p* < 0.001	r_s_ = − 0.266 *p* < 0.001
Moderate, poor, very poor, *median (IQR)*	33.75 (26.25–43.75)	5 (2.5–6.25)	7.5 (5–11.25)	3.75 (1.25–7.5)	52.5 (41.25–65_
Very good or good, *median (IQR)*	27.5 (20–35.635)	3.75 (1.25–6.875)	6.25 (5–8.75)	1.25 (0–5)	42.5 (28.125–52.5)
Mann–Whitney test	z = 3.617, *p* < 0.001	z = 1.136, *p* = 0.258	z = 2.183, *p* = 0.029	z = 4.172, *p* < 0.001	z = 3.787, *p* < 0.001
ES	r = 0.222	r = 0.070	r = 0.134	r = 0.256	r = 0.233
*Types of diabetes*					
Type I, *median (IQR)*	30 (25–38.75)	3.75 (1.25–6.25)	6.25 (5–8.75)	2.5 (1.25–6.25)	47.5 (31.25–56.25)
Type II, *median (IQR)*	31.25 (23.75–41.875)	5 (2.5–6.875)	7.5 (5–10.625)	2.5 (0.625–6.25)	47.5 (36.25–62.5)
Mann–Whitney test	z = − 0.681, *p* = 0.499	z = − 0.767, *p* = 0.446	z = − 1.960, *p* = 0.050	z = − 0.265, *p* = 0.794	z = − 0.874, *p* = 0.385
ES	r = − 0.043	r = − 0.048	r = − 0.123	r = − 0.017	r = − 0.055
*Years since diagnosis, Spearman’s correlation*	r_s_ = 0.087 *p* = 0.155	r_s_ = 0.084 *p* = 0.171	r_s_ = 0.019 *p* = 0.763	r_s_ = 0.140 *p* = 0.022	r_s_ = 0.100 *p* = 0.104
*Years of duration of diabetes*					
Up to 2 years, *median (IQR)*	31.25 (21.25–37.5)	3.75 (1.25–6.25)	7.5 (5–10)	2.5 (0–5)	45 (33.75–57.5)
3 to 5 years, m*edian (IQR)*	32.5 (24.375–41.25)	3.75 (2.5–6.25)	8.75 (6.25–10)	2.5 (1.25–5)	50 (38.125–61.875)
6 or more years, *median (IQR)*	32.5 (25–42.5)	5 (2.5–7.5)	7.5 (5–10.625)	5 (1.25–7.5)	48.75 (38.125–63.75)
Kruskal Wallis H	H(2) = 2.853, *p* = 0.240	H(2) = 2.681, *p* = 0.262	H(2) = 1.581, *p* = 0.454	H(2) = 4.293, *p* = 0.117	H(2) = 3.056, *p* = 0.217

*SD* standard deviation, *IQR* interquartile range, *ES* effect size

^a^The total PAID score is the sum of the 20 items multiplied by 1.25. The four scales of the Spanish model are calculated in a similar way

### Exploratory factor analysis

An EFA was conducted to examine the structure further. Forced EFAs revealed four factors (eigenvalues above 1). As in the Spanish model, two items loaded on a treatment-problems factor, while three items loaded on a social-support-problems factor. Nevertheless, the other two factors were not as homogeneous as in the Spanish model. The three items related to food problems loaded highly on a single factor. Nonetheless, some of the items from the emotional-problems subdimension also loaded on this factor, rendering the interpretation of these two factors equivocal.

### Internal consistency

Cronbach’s α (0.88) and composite reliability (0.91) were good for the full scale. However, only the scale of emotional problems of the four-factor Spanish structure yielded a good Cronbach’s α and composite reliability above 0.8 (Table 4).

## Discussion

We translated and culturally adapted the PAID questionnaire into Kinyarwanda. To our knowledge this is the first study to provide a cross-culturally adapted and psychometrically evaluated version of a diabetes-specific distress questionnaire in Rwanda. The full scale showed a good internal reliability in line with previous studies [8, 15]. The four-factor model previously tested in Spain showed an adequate approximate fit of the data [20].

Consistent with the results of other studies [8, 14, 19, 20, 22, 39], *“worrying about the future and the possibility of serious complications”* was the question with the highest reported distress (mean = 3.11, SD = 1.15; median = 4, IQR = 3–4): it was perceived as a somewhat serious or serious problem by 77.4% of the participants. Nonetheless, the other items receiving high scores were *“feeling overwhelmed by your diabetes”* (mean = 2.39, SD = 1.33; median = 2, IQR = 1–4; 49.8% of the participants endorsed it as a somewhat serious or serious problem) and *“feeling constantly concerned about food and eating”* (mean = 2.38, SD = 1.39; median = 3, IQR = 1–4; 50.6% of participants endorsed it as a somewhat serious or serious problem), indicating different problem areas compared with other cultures.

Dissatisfaction with one’s diabetes physician was considered the least distressing item (mean = 0.54, SD = 1.14; median = 0, IQR = 0–0; 77.1% of the participants reject the idea of this being a problem) in concordance with the research of Polonsky et al. in the United States and Huang et al. in Taiwan [14, 39]. Interestingly, in previous studies this item also yielded a low mean score, however, not as low as the perceived distress deriving from the lack of support of one’s diabetes management efforts by friends and family [19, 21]. The effect of the setting and the presence of health care providers may explain the result in Rwanda, as participants completed the questionnaire in nurse-run clinics for non-communicable diseases in hospitals [14].

The mean total PAID score in our study was notably high (48.21). One hundred eighty-three (68.8%) had a total score of 40 or higher, which suggests severe diabetes-specific emotional problems according to a previously designated cut-off score [7, 8]. Cross-cultural differences in the PAID score have been observed elsewhere [3, 10, 19]. In the study of Snoek et al. [19], the Dutch sample showed lower distress compared with the demographically- and clinically-comparable American one, although both populations regarded similar problem areas as the most distressing. Ogbera and Adeyemi-Doro [10] documented a mean PAID score of 21.3 in patients with type 2 diabetes mellitus in Lagos. Spencer et al. [3] identified a significant difference between inner-city African American and Hispanic patients with type 2 diabetes mellitus in Detroit (mean scores of 15.59 vs. 36.75 respectively). Melkus et al. [40] found a mean PAID score of 49.3 in a small sample of African American women. Significant differences on distress have also been observed in ethnic minorities in the Netherlands [41, 42]. It should be noted, lastly, that the cut-off was established in Western populations (one standard deviation above the mean) [2, 43] and may not be universally applicable.

The observed higher PAID score may be partially explained by the coexistence of general emotional distress, including depression and anxiety, the ways life and environmental stressors are perceived and prioritised, and the temperament [3, 44, 45]. Depression and diabetes-specific distress are substantially overlapping constructs, with common symptoms and presentations [4, 7]. The questionnaire and some of its items have shown a strong correlation with several related non-disease-specific constructs [15]. The performance of the questionnaire as a screening tool for clinical and subclinical depression was documented in Germany [7]. A recent study in Rwanda screened patients with type 2 diabetes mellitus for depression using the Patient Health Questionnaire-9 (PHQ-9) and reported minimal to severe depression in 83.8% of the sample. The authors suggested the possibility of linking such a high prevalence to the 1994 Genocide against the Tutsi, as the population had been more exposed to general emotional distress [46]. Paucity of health care professionals and poor access to health care services as a result of the Genocide should also be considered [47]. Yet, it is important to distinguish depression from diabetes-specific distress, for they can present alone or simultaneously, and their coexistence can be inter-dependent or not. Notably, diabetes-specific distress has been shown to be an independent predictor of impaired glycaemic control and poor self-care [2, 7, 14].

Another indicator of cross-cultural differences in experiencing diabetes-specific distress is the wide range of reported factorial structures, with some studies confirming the unidimensional original model and others suggesting up to four factors [8, 18–21, 23, 24, 39]. While many studies observed one large emotional-problems factor [18–21], the food-related factor was not always distinct [23, 24]. The Spanish model, which yielded the best approximate fit, differs from the Dutch model only in integrating the item *“feeling unsatisfied with your diabetes physician”* into the factor of social-support problems [19, 20]. Other studies regarded this item as a social-support problem too [23, 48]. All these findings suggest caution concerning the cross-culture applicability of PAID [48].

Unlike other studies [4, 12, 19], but in accordance with the results of Ogbera and Adeyemi-Doro [10], there was no link between female gender and diabetes-specific distress. There was a small effect of the type of residency area, with participants coming from more rural areas reporting higher distress levels. Moreover, a weak inverse correlation was observed between the years of completed education and the total PAID score. Having no formal education or having completed primary school was related to higher distress. A decreased ability of coping with the management of the diseases may be linked to these results [49].

The presentation of diabetes-specific distress may differ between patients with type 1 and type 2 diabetes mellitus. For instance, the former may present higher fear of hypoglycaemia [4]. Although we observed no significant differences between the two types, our sample consisted predominantly of patients with type 2 diabetes mellitus. Such a distinction should be considered with caution on the grounds of possible misclassification and possible atypical disease forms [50–53]. There may also be a separate effect of the treatment type, specifically related to the use of insulin [21], however information on insulin use was not collected in the present study.

An additional study limitation was the lack of testing for convergent validity, as it was not possible to identify another established and previously validated generic or diabetes-specific tool in the Rwandan population. Test–retest reliability was not carried out, and further research is therefore needed in this respect. Moreover, we were unable to evaluate the correlation of PAID with HbA1c, as this test was not yet performed in patients with diabetes systematically and we could not carry it out ad-hoc for the entire study sample. Finally, while efforts were made to investigate comorbidity, it was difficult to obtain reliable diagnoses from the medical records of the patients. Yet, hypertension, which was easier to verify, was estimated at 42.48% of the sample and had a small effect on distress (z = 2.240, *p* = 0.025, r = 0.137).

### Conclusions

The PAID questionnaire is brief and easy to administer and could help both researchers and clinicians to identify appropriate interventions and targeting at-risk populations. We could show important differences between its Kinyarwanda version and those studied on other populations, such as the way disease-specific distress was experienced and the factors that cause the most distress [8, 14, 19, 20, 22, 39]. These results urge further examination of cultural differences in the questionnaire’s underlying concepts.

## Data Availability

The data used to support the findings of this study are restricted by the Government of Rwanda and cannot be released or shared partially or totally with third parties without the written permission of the Rwanda Biomedical Center. Data are available from the corresponding author for researchers who meet the criteria for access to confidential data, and only after authorisation from the Rwanda Biomedical Center.

## Appendix: The Kinyarwanda version of the problem areas in diabetes (PAID) questionnaire

INGORANE ZIKOMOKA KURI DIYABETE

AMABWIRIZA: Ni iyihe muri izi ngorane za diyabete zikurikira iguteye *ikibazo muri iki gihe*?

Shyira mu ruziga umubare utanga igisubizo kibereye kuruta ibindi kuri wowe. Tanga igisubizo kuri buri kibazo.

**Table:**

INGORANE	Nta kibazo	Ikibazo gito	Ikibazo kiringaniye	Ikibazo gisa n'igikomeye	Ikibazo gikomeye
1. Kutagira intego zisobanutse kandi zifatika mu kwiyitaho kuri diyabete yawe?	0	1	2	3	4
2. Kwiyumvamo gucika intege mu gukurikiza gahunda za buri gihe z'ivurwa rya diyabete yawe?	0	1	2	3	4
3. Kwiyumvamo ubwoba kubera gutekereza ko ufite diyabete?	0	1	2	3	4
4. Ingorane mu mibanire rusange ziva mu kwita kuri diyabete yawe? (urugero: nko kubona abantu bakubwira ibyo ugomba kurya)	0	1	2	3	4
5. Kwiyumvamo kwiyima ku bijyanye n'ibiribwa n'indyo?	0	1	2	3	4
6. Kumva wihebye iyo utekereje ko ufite diyabete?	0	1	2	3	4
7. Kuba utazi niba uburyo wiyumvamo buterwa na diyabete yawe?	0	1	2	3	4
8. Kubuzwa amahwemo na diyabete yawe?	0	1	2	3	4
9. Guhangayikishwa n'ingaruka zazanwa n'igabanuka ry'ibipimo by'isukari iri mu maraso?	0	1	2	3	4
10. Kugira uburakari iyo utekereje ko ufite diyabete?	0	1	2	3	4
11. Guhora uhangayikishijwe n’ibyo urya?	0	1	2	3	4
12. Guhangayikishwa n'ejo hazaza hawe n'uko hari ibindi bibazo by'uburwayi bikomeye byaturuka kuri diyabete?	0	1	2	3	4
13. Kumva wishinja no guhangayika iyo utaye umurongo ku bijyanye no gufata neza diyabete yawe?	0	1	2	3	4
14. “Kutemera” na diyabete yawe?	0	1	2	3	4
15. Kumva utanyuzwe na muganga ukuvura diyabete?	0	1	2	3	4
16. Kumva ubujijwe amahwemo bikomeye na diyabete mu mutwe no mu mubiri buri munsi?	0	1	2	3	4
17. Kumva ko uri wenyine kubera diyabete yawe?	0	1	2	3	4
18. Kumva inshuti n'umuryango batagushyigikira mu buryo uharanira kwiyitaho mu burwayi bwa diyabete?	0	1	2	3	4
19. Guhangana n'ibindi bibazo by'uburwayi bituruka kuri diyabete?	0	1	2	3	4
20. Kwiyumvamo ko urambiwe cyane kubera imbaraga zisabwa iteka mu gucunga diyabete?	0	1	2	3	4

## Abbreviations

CFA: Confirmatory factor analysis
CFI: Comparative fit index
DF: Degrees of freedom
EFA: Exploratory factor analysis
ES: Effect size
HbA1c: Haemoglobin A1C
IQR: Interquartile range
PAID: Problem areas in diabetes
RCT: Randomised controlled trial
RMSEA: Root mean square error of approximation
SD: Standard deviation
TLI: Tucker Lewis index
WLSMV: Weighted least square mean and variance
WRMR: Weighted root mean-square residual
